# Fluorescence in situ hybridization as adjunct to cytology improves the diagnosis and directs estimation of prognosis of malignant pleural effusions

**DOI:** 10.1186/1749-8090-7-121

**Published:** 2012-11-13

**Authors:** Jingquan Han, Shouqiang Cao, Kai Zhang, Guibin Zhao, Yanzhong Xin, Qing Dong, Yubo Yan, Jian Cui

**Affiliations:** 1 Department of Thoracic Surgery, The Fourth Affiliated Hospital, Harbin Medical University, No.37, Yi yuan Street, Nan gang District, Harbin, Heilongjiang Province, 150001, China

**Keywords:** Malignant pleural effusions, FISH, Prognosis, Sensitivity, Specificity

## Abstract

**Background:**

The identification of malignant cells in effusions by conventional cytology is hampered by its limited sensitivity and specificity. The aim of this study was to investigate the value of fluorescence in situ hybridization (FISH) as adjuncts to conventional cytologic examination in patients with malignant pleural effusions.

**Methods:**

We conducted a retrospective cohort study of 93 inpatients with pleural effusions (72 malignant pleural effusions metastatic from 11 different organs and 21 benign) over 23 months. All the patients came from Chinese northeast areas. Aspirated pleural fluid underwent cytologic examination and fluorescence in situ hybridization (FISH) for aneuploidy. We used FISH in single-colour or if appropriate in dual-colour evaluation to detect chromosomal aberrations (chromosomes 7, 11, and 17) in effusion cells as markers of malignancy, to raise the diagnostic yield and identified the efficiency by diagnostic biopsy. Predominant cytogenetic anomalies and patterns of intratumor cytogenetic heterogeneity were brought in relation to overall survival rate.

**Results:**

Cytology alone confirmed malignant pleural effusions in 45 of 72 patients (sensitivity 63%), whereas FISH alone positively identified 48 of 72 patients (sensitivity 67%). Both tests had high specificity in predicting benign effusions. If cytology and FISH were considered together, they exhibited 88% sensitivity and 94.5% specificity in discriminating benign and malignant effusions. Combined, the two assays were more sensitive than either test alone. Although the positive predictive value of each test was 94.5%, the negative predictive value of cytology and FISH combined was 78%, better than 47% and 44% for FISH and cytology alone, respectively. There was a significantly prolonged survival rate for patients with aneuploidy for chromosome 17.

**Conclusions:**

FISH in combination with conventional cytology is a highly sensitive and specific diagnostic tool for detecting malignant cells in pleural effusions . The high sensitivity and specificity may be associated with geographic area and race. Simple numeric FISH anomalies may be prognostic.

## Background

Diagnosis of malignancy in pleural effusions from cancer patients is frequently troublesome for the cytologist because the differentiation of malignant cells from accompanying elements such as atypical reactive mesothelial cells is difficult [1]. On the other hand, in pleural effusions tumour cells may appear quite similar to normal cells, for example, small-cell lung cancer cells and lymphocytes [2]. Due to these difficulties, cytopathologists traditionally adopt a rather cautious approach in the diagnosis of malignancy in effusions. With a negative cytology result in a patient with suspected malignant pleural effusion, standard clinical practice involves a repeat thoracentesis, a pleural biopsy, or thoracoscopy to prove the presence of tumor cells in the pleural fluid. Thoracoscopy is effective in this regard and has a high diagnostic yield for malignant disease involving the pleura [3]. Nevertheless, the risks of additional diagnostic procedures that are painful and invasive, such as thoracoscopy, present a considerable clinical challenge. Then new methods complementing cytology for the diagnostic work-up of pleural effusion need to be evaluated.

Genomic alterations are a hallmark of malignant cells. In the resent years, interphase cytogenetics by fluorescence in situ hybridization (FISH) has been increasingly used in clinical pathology to delineate chromosomal aberrations in neoplasia [4-8]. Because carcinoma cells are regularly characterized by extensive chromosomal aberrations, FISH analysis can be used as a diagnostic tool to detect aneuploid cells characterizing malignancy, provided that cutoffs for background nondisomy are considered. We performed tumour-associated aneuploidy analyses on prospectively collected pleural fluids in order to investigate if there was a complimentarity of cytology and chromosomal aberrations assays in detecting malignant tumor cells in the fluids. Overall survival rate from the first diagnosis of malignancy until death or final follow-up was evaluated. We hypothesized that FISH would be able to detect patients with malignant chromosomal aberrations in their pleural fluid and that, used with cytology, they would enhance diagnosis. Moreover, FISH may be associated with survival rate in patients with malignant pleural effusions.

## Methods

### Clinical and pathologic features of the study population

Effusion sampling and investigation analyses had been approved by the institutional ethics committee. The study population comprised 93 patients with documented pleural effusions who underwent pleural fluid aspiration for diagnostic purposes at The Fourth Affiliated Hospital of Harbin Medical University between October 2008 and September 2011. All the patients were born in Chinese north areas. The study was presented to the Hospital Ethical Board and accepted as this is an observational study based on the best available evidence. The research was conducted conformed to the Helsinki Declaration and to local legislation. Patients gave informed consent to participate in the study. Results of conventional cytology and FISH analyses of the pleural fluids from patients were compared with respect to the definitive diagnosis established by either tissue biopsy, or through clinical follow-up in the case of patients with benign disease. Patients who were free of malignancy had a median follow-up of 6.5 months (range, 1.2 to 32.4). The median age of the study population was 68 years (range, 23 to 85) and there was a predominance of males (63 of 93, or 68%). Pleural invasion was defined as invasion of tumor through either the parietal or visceral pleura.

Pleural fluid was obtained either via a needle during thoracentesis (42%), a chest tube during thoracostomy (13%) or by aspiration through a 1-cm incision at the very beginning of a thorascopic pleurodesis procedure (45%). Aspirated pleural fluid was collected in sterile tubes without anticoagulant and rapidly brought to The Fourth Affiliated Hospital of Harbin Medical University pathology laboratory for conventional cytologic examinations as well as to the research laboratory where they were analyzed by FISH.

### Conventional cytology

About 100 cc of aspirated pleural fluid was spun in a centrifuge at 2,160 rpm for 10 minutes, supernatant removed, and the cell pellet both preserved onto glass slides in ethanol for Papnicolau staining as well as in formalin and ethanol for processing into cellblocks as per standard protocol in the hospital pathology laboratory. The presence or absence of malignant cells in the cytologic material was reported with the agreement of two attending cytopathologistes.

### Fluorescence in situ hybridisation analysis

Cells of at least 200 mL of effusion fluid were gained by centrifugation, and, in case of macroscopic blood contamination, subjected to density gradient separation over Ficoll-Hypaque (Sigma, St Louis, MO). Pelleted effusion cells were washed in phosphate-buffered saline, fixed in methanol-acetic acid (3:1, v/v) and stored at −80°C. Directly fluorescence-labeled alpha-satellite DNA probes (SpectrumGreen [excitation peak of 497 nm, emission peak of 524 nm] and SpectrumOrange [559 nm/588 nm]; Vysis Inc, Downers Grove; IL) were applied in dual-color FISH experiments. The probes used in this study were specific for the centromeres of chromosomes 7, 11, and 17. The standard protocol followed was described in a previous report [9].

### Fluorescence microscopy and definition of cutoffs for aneuploidy

A fluorescence microscope with × 60 and ×100 planar objectives and appropriate filter sets was used for FISH signal evaluation and documentation. All effusion cells in a field except for polynucleated granulocytes, which are easily distinguishable by nuclear shape, were analysed. The stringent criteria of FISH signal assessment were applied to avoid overestimation of hyperdiploidy, which may result from cellular and technical factors [10]. Signal counting was performed by two investigators and intraobserver, and interobserver counting variations were evaluated repeatedly. In order to evaluate the frequencies of aneusomic effusion cells with statistical reliability, centromeric signals of 100–1000 nuclei were scored, with high-number cell counting in samples with a low frequency of aneuploidy [11]. When necessary, we used a two-tiered scoring procedure: (1) in all 93 effusions, scoring of nuclei in single-colour FISH evaluation was performed, and if aneusomy above cutoff for any of the tested chromosomes was present, malignancy could be documented; otherwise, in step (2), scoring of selected, namely hyperdisomic, nuclei was performed in dual-colour FISH evaluation, which allowed for the detection of rare FISH-aneuploid cells [12]. For unequivocal identification of true aneuploidy representing malignancy, stringent cutoff threshold levels for background nondisomy were applied. The resulting mean percentages of aneusomy and standard deviations were used to calculate chromosome-specific background cutoff levels for aneusomy, which were applied in the study presented here (Table 1). The mean percentages plus 3 standard deviations of control cells with 1, 3, 4, and greater than 4 signals were taken as cutoffs (Table 1). Test effusions were considered as aneuploid when the percentages of nondiploid nuclei (categorized into cell populations with 1, 3, 4, greater than 4 signals) exceeded respective cutoff levels. In cases in which the diagnosis of aneuploidy was based on a rate of combined aneuploidy in less than 5% of cells, a repeated FISH experiment using appropriate probes for reconfirmation was performed.

**Table 1:** **Cutoff threshold levels for background aneusomy** (**percentage**) **for the three chromosomes examined**

	**Signal number category**			
	**1**	**3**	**4**	**>4**
Chromosome 7	11.5	0.6	0.7	0.2
Chromosome 11	11.2	0.4	1.2	0.2
Chromosome17	13.4	0.9	1.0	0.1

The presented cutoffs were determined by evaluation of non-disomy in 15 nonmalignant control effusions, and reflect the mean+3 s.d. percentage of aneusomic cells within each of four FISH signal categories.

### Statistical analysis

Sensitivity, specificity, positive predictive values and negative predictive values of both FISH and conventional cytology as well as of the combination of both techniques were calculated in relation to the definitive diagnosis of the patients in the study. Correlation between variables was estimated using the Fisher’s exact test, or the χ^2^ test when appropriate. Survival time was analyzed using the Kaplan-Meier method, and differences between two groups(exhibiting or not exhibiting aneuploidy for chromosome 17) were evaluated with the log-rank test. All *P* values are two-sided, and all significant associations were considered when the *P* value was 0.05 or less. SSPS16.0 software was used for calculations.

## Results

Of the 72 patients with malignancy as the definitive diagnosis, the most frequent underlying tumour entities were lung, breast, colon, ovarian (Table 2). Primary cancers from the lung and breast represented the overwhelming majority of malignancies: 48 of 72 (67%).

**Table 2:** Definitive Diagnosis of Patients in Study

**Type of Primary Cancer**, **n**=**93**	**No. (%)**
Nonsmall cell lung	39 (41.9)
Breast	9 (9.7)
Colon	6 (6.5)
Ovarian	5 (5.4)
Gastric	3 (3.2)
Haematological	2 (2.2)
Renal	2 (2.2)
Mesothelioma	2 (2.2)
Skin	2 (2.2)
Chrondrosarcoma	1 (1.1)
Cervical	1 (1.1)
Benign diseae	21 (22.6)

### Sensitivity, positive predictive value, and negative predictive value of conventional cytology, fluorescence in situ hybridisation, and the tests combined

**Figure 1:**
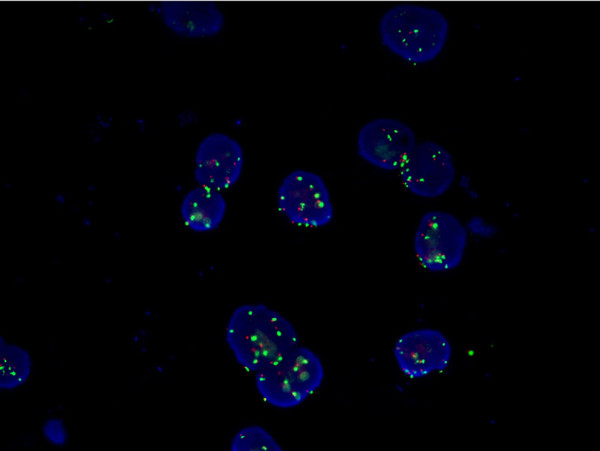
**Malignant pleural effusion(non**-**small cell lung cancer)with dual-color FISH-stained tumor cells showing differing distribution patterns of green and orange signals(chromosomes 7 and 17,respectively),indicative of intranuclear chromosomal complexity.**

Conventional cytology alone detected the presence of neoplastic cells in 45 of 72 patients (sensitivity of 63%) whereas no malignant cells were isolated from the pleural fluid of benign patients (specificity of 90.2%; *P*=0.006). Fluorescence in situ hybridisation analysis was performed in single-colour FISH evaluation when aneusomy was present above a cutoff value, unambiguously discriminating tumour-associated aneusomy from background ‘physiological’ aneusomy. When aneusomic cells were rare, evaluation in dual-colour FISH evaluation was performed aiming at discriminating polyploidy from aneuploidy originating from tumour cells (Figure 1). Fluorescence in situ hybridisation analysis alone was diagnostic for malignant cells in 48 out of 72 effusions (sensitivity of 67%) while no patient with benign disease had a positive test of their pleural fluid (specificity of 98.7%; *P*=0.002). If conventional cytology and FISH were considered together, the two techniques showed complementarity increasing the sensitivity to 87.5% while maintaining a specificity of 94.5% in discriminating benign and malignant disease. The positive predictive value for FISH alone, cytology alone, as well as the combination of both techniques was over 90%. The negative predictor value of cytology and FISH tests alone were 44% and 47%, respectively. The combination of both techniques increased the negative predictor value to 78%.

### Correlation of cytology and FISH analyses of pleural fluid

Although both cytology and FISH analysis were comparable in terms of sensitivity, the tests were independent of each other. FISH identified 30 of 45 pleural fluid aspirates (67%) that were positive by cytologic analysis. In addition, Fluorescence in situ hybridisation detected the presence of chromosomal aberrations in the fluid from 18 of 27 patients (67%) who had cytology negative aspirates. In 9 patients (1 patient with acute myelocytic leukemia, 1 with renal cell carcinoma, 2 with ovarian carcinoma, 2 with skin carcinoma, and 3 with NSCLC), cytology and FISH as a combined analysis failed to detect malignant cells or chromosomal aberrations, respectively. FISH had a better sensitivity in predicting the presence of a malignant pleural effusion from a NSCLC primary cancer than cytology (30 of 39, or 77%, versus 24 of 39, or 62%, respectively). Of those patients with breast cancers, cytology and FISH both were able to detect malignant pleural effusions with sensitivities of over 90%.

### Transudative versus exudative pleural fluid

Data concerning the character of the pleural effusions (transudative or exudative) was available on 69 patients (74%). A transudate was defined by a protein concentration of <2.5 g dl^-1^[13]. There were six transudative effusions in the patients with a primary malignancy and both cytology as well as molecular examination did not discern in these specimens any malignant cells or chromosomal aberrations respectively. There were also 51 exudative effusions in all, 9 of which were in patients with no malignancy. The combined tests of FISH and cytology were more accurately able to classify the exudative effusions as being malignant (33 of 42, or 79%) than either cytology (24 of 42, or 57%) or FISH alone (27 of 42, or 64%).

### Analysis of prognosis of patients with malignant pleural effusions

**Figure 2:**
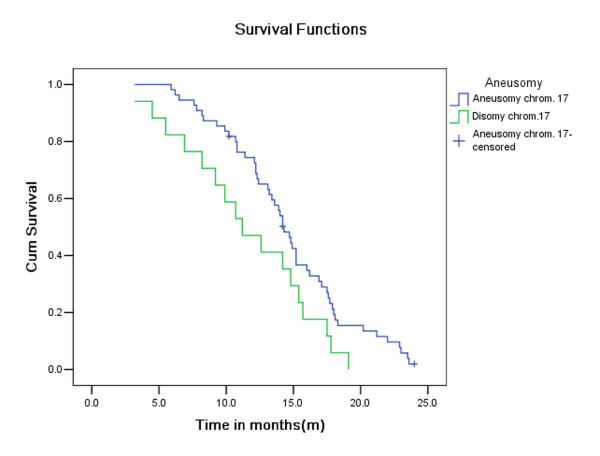
**Overall survival rate of patients with effusion cells exhibiting or not exhibiting aneuploidy for chromosome 17.** There was a significantly prolonged survival rate for patients with aneuploidy for chromosome 17, which was always a gain of copy number except for one patient who exhibited predominantly monosomy 17.

As shown in Figure 2, the overall two-year survival rates for patients without exhibiting aneuploidy for chromosome 17 (76.4%; 55 of 72) were lower than those for patients with exhibiting aneuploidy for chromosome 17 (25.6%; 17 of 72). This difference was statistically significant (*P* =0.04; log-rank test).

## Discussion

Molecular analysis of the pleural fluid of patients using fluorescence in situ hybridisation to discriminate those with benign and malignant pleural effusions has not been well studied. We hypothesized that in patients in whom malignant cells may not be detectable in the pleural fluid by conventional cytology, tumour-associated aneuploidy would be present in sufficient quantities to be detected, and that the sensitivity of this approach would improve the overall diagnostic sensitivity of conventional cytology. Our data show that even for multiple tumor types, and with a limited cohort size, we were able to improve the diagnostic performance of cytologic analysis by measuring tumour-associated aneuploidy in the pleural fluid. We employed a molecular technique and observed that conventional cytology and FISH analysis alone have similar sensitivities (63% and 67%, respectively), but that the sensitivity improves to 88% when the results of the FISH and cytology techniques are combined. It is noteworthy that cytology analysis cost $60 for each case vs $625 of cytology analysis combined with FISH. It has been proposed that the use of tissue microarray for FISH might provide satisfactory results with a markedly cost reduction.

Tumor cell detection in effusions can be significantly improved by FISH and PCR techniques applying appropriate molecular markers. PCR method targeted at tumor-specific genetic abnormalities could detect a small number of cancer cells mixed within a large number of normal cells. This method offered considerable promise for early diagnosis of malignant pleural effusions and detection of tumor progression before clinically evident metastasis. However, the clinical application has been limited so far. The primary limitation had been the lack of suitable target genes common to most tumors. The other limitation of RT-PCR is related to laboratory method per se.

FISH analysis for the diagnosis of malignancy is based on the fact that tumor cells are regularly chromosomally aberrant, mostly harboring complex polysomies for one or multiple chromosomes. The methodical approach used here is based on the previous observation that the identification and quantification of aneuploidy by FISH can be used as a sensitive and highly specific marker of malignancy in metastatic cells [11,14-17]. We selected chromosomes 7, 11, and 17 for this study, which were found to be prognostic markers in malignant tumour [18-21]. We chose to use a combination of probes to these 3 chromosomes as the basis for our FISH test to detect genetically abnormal pleural effusions. Although we had tumors from 11 different organ sites, we designed a broad panel of genes to detect primarily breast and lung tumors since the most common malignancies that metastasize to the pleural fluid are, in order of frequency, lung and breast. Our chromosome panel was successful in detecting breast and lung neoplasms with sensitivities of 100% and 75%, respectively. Moreover, 15 of the 24 tumors that FISH alone failed to detect were not covered by our panel, namely, 1 patient with acute myelocytic leukemia, 1 with renal cell carcinoma, 1 with cervical, 1 with chrondrosarcoma, 2 gastric,2 with ovarian, 2 with skin, 2 with colon and 3 with NSCLC. Indeed, it will be a challenge to be borne out of subsequent studies to determine how large of a panel of chromosomes will be required to identify the variety of neoplasms that metastasize to the pleural cavity.

The survival rate correlated predominant aneusomy for chromosomes 11 has been previously reported in breast cancer and NSCLC. For breast cancer, patients with no exhibiting aneuploidy for chromosome 11 in cancerous effusions had a significantly shorter overall survival rate than did have patients who had aneuploidy [18]. Other studies also found that poor prognosis was associated with aneusomy of chromosome 17 in breast cancer [22,23]. In the present work, our results were consistent with the above-mentioned studies showing that aneuploidy for chromosome 17 was associated with poor prognosis in patients with malignant pleural effusions. But survival rate was not found to correlate with the predominant aneusomy for chromosomes 7 and 11.

We also observed in the present study that pleural fluids positive by the combined FISH and cytology analysis correlated with pleural invasion. These data suggest that there is a higher yield of malignant cells in the pleural fluid after the pleura is invaded by tumor. This is in agreement with recent studies performed using pleural lavages during pulmonary resections which have shown increased detection of positive pleural lavage cytology when there is parietal pleura invasion by malignancy [24]. Combined cytology and FISH analysis were also more accurate in discerning exudative samples from patients with a primary malignancy. In general, exudative malignant pleural effusions have higher cell counts, lower glucose and pH levels, and are cytologically positive. But these criteria are far from absolute [25], and in our dataset, cytologic analysis only detected 57% of exudative pleural fluids from patients with a known primary malignancy compared with 64% for FISH alone and 79% for the chromosome and cytology assays combined.

Limitations of the present study include the small cohort size that precludes detailed analysis of specific tumor subtypes. Only descriptive, observational conclusions about the FISH analyses in patients with NSCLC and breast cancer can be made. Second, only FISH technology with 3 probes were used in this study. The patients mainly came from Chinese north areas. Therefore, it is need more study in a large patient group and varieties areas in China in order to figure out specific chromosome anomalies to specific tumors in large population. Finally, the patients with benign disease in this study only had 6 months of longitudinal follow-up to determine if they remained cancer free. That is usually insufficient time to allow preneoplastic events to progress to clinically detectable cancer. Despite our ability to detect tumour-associated aneuploidy in pleural fluid, the sensitivity of this assay would most likely be enhanced by designing separate chromosome panels for tumors originating from specific organs. Chromosome profiles of tumors from different organs do seem to be distinct [26,27], but more validity studies are necessary before these profiles can be optimized for routine clinical diagnostic use. Improving the diagnostic performance of any test has the potential to alter medical practice, even if, as in this case, it simply means eliminating the need for repeated thoracentesis or other invasive procedures to secure the diagnosis of metastatic disease in a patient with a know primary tumor. Although a more sensitive test to diagnose patients earlier may have limited clinical value in extending survival in patients with metastatic disease, this assay may have important diagnostic implications particularly in patients who have unilateral pleural effusions as their principal presenting clinical sign. It is conceivable that chromosome profiles of the pleural fluid could also be used to identify the site of origin of the metastatic malignant cells in the chest. But this technology requires much validation before it becomes clinically widespread.

## Conclusion

In conclusion, this study shows the feasibility of detecting tumour-associated aneuploidy from a variety of different malignancies in the pleural fluid of patients even if conventional cytologic analyses are negative. In addition, the FISH test may increase the sensitivity of conventional cytology without a corresponding decrease in specificity. The present results were obtained retrospectively and suggest that simple numeric FISH anomalies may be prognostic. Gainning of chromosome 17 in malignant pleural effusions is a marker of superior prognosis. This observation may prompt us to perform prospective studies in which chromosomal changes in malignancies will be correlated with survival rate. Larger translational studies will be needed to validate that molecular markers may improve the diagnostic yield of the current standard examination of pleural fluid.

## Abbreviation

FISH: Fluorescence in situ hybridization.

## Competing interests

The authors declare that they have no competing interests.

## Authors’ contributions

JH and SC conceived the study and drafted the manuscript. JH, SC, KZ, GZ, YX and YY performed the experiments. QD analyzed the data. JC supervised the experimental work. All authors have read and approved the final manuscript.
